# Distributed retrieval engine for the development of cloud-deployed biological databases

**DOI:** 10.1186/s13040-018-0185-5

**Published:** 2018-11-12

**Authors:** David Bouzaglo, Israel Chasida, Elishai Ezra Tsur

**Affiliations:** Neuro-biomorphic Engineering Lab, Faculty of Engineering, Jerusalem College of Technology, Jerusalem, Israel

**Keywords:** MongoDB, Specialized databases, Federated databases, Cloud-based databases

## Abstract

The integration of cloud resources with federated data retrieval has the potential of improving the maintenance, accessibility and performance of specialized databases in the biomedical field. However, such an integrative approach requires technical expertise in cloud computing, usage of a data retrieval engine and development of a unified data-model, which can encapsulate the heterogeneity of biological data. Here, a framework for the development of cloud-based biological specialized databases is proposed. It is powered by a distributed biodata retrieval system, able to interface with different data formats, as well as provides an integrated way for data exploration. The proposed framework was implemented using Java as the development environment, and MongoDB as the database manager. Syntactic analysis was based on BSON, jsoup, Apache Commons and w3c.dom open libraries. Framework is available in: http://nbel-lab.com and is distributed under the creative common agreement.

**Electronic supplementary material:**

The online version of this article (10.1186/s13040-018-0185-5) contains supplementary material, which is available to authorized users.

## Background

The growing rate of biological data generation has produced unprecedented data streams, which regularly renovate our understanding of system biology [1], as well as alter our practice in healthcare [2]. As biomedical research became transdisciplinary, data across numerous levels of granularities and perspectives has to be acquired and integrated. Moreover, for meta-analysis, data is often gathered from multiple archival databases. To mitigate the growing rate of biodata origination, new frameworks for data acquisition, classification, storage, retrieval and analysis are being developed continuously.

Among the challenges underlying many such frameworks are the heterogeneity of biological data types and the emergence of new relations between data entities [3]. As a result, on top of the traditional primary and secondary databases, specialized databases were developed. Specialized databases include organism-centered datasets [4, 5], biological pathways [6] and diseases [7], each with its own data specifications, often curated to serve consortiums or single laboratories. Specialized databases often integrate data from numerous primary, secondary and other specialized databases.

The necessity of finding a piece of data in a vast array of databases led to the development of meta search engines, which are traditionally based on a distributed approach. A distributed search engine is a decentralized service, allocating mining and query generation among numerous edges, integrating the retrieved results in a unified framework, constituting a federated database. For example, the Neuroscience Information Framework (NIF) [8], one of the most important database federations for the neuroscience community, has been cataloging and surveying the neuroscience resource landscape since 2006. NIF currently gives access to over 250 data sources categorized to different subjects ranging from software tools to funding resources. NIF provides a distributed query engine to specialized data bases, which are independently created and curated. This type of distributed search among independent databases is enabled through NIF’s DISCO registry tool with which a Web resource can send both automatic or manual data updates to the NIF system [9].

One of the most important stepping stones in modern biodata mining is cloud computing, providing scalable virtualized resources and distributed computing, and enabling optimization of cost and computing efficiency [10]. Thus, cloud providing frameworks, such as IBM Cloud [11], Microsoft Azure [12], Amazon AWS [13] and Google Cloud [14] are routinely adopted by research groups and organizations. Cloud-based federated data-bases can provide powerful framework for integrated data-centered research. For example, Todor and colleagues developed the ChemCloud, a semantic-web based framework, which integrates specialized local databases with online datasets in the fields of chemistry and pharmacy, aiming at semantic search, semantic data enrichment, ontology-enhanced navigation, machine generated eLearning trajectories and semantic knowledge discovery over multiple databases [15]. O’Connor and colleagues developed the SeqWare, a database framework aiming at handling and querying a wide range of genomic-related data types, utilizing the Hadoop MapReduce environment and the Hadoop HDFS distributed filesystem [16].

Integrating cloud resources and federated data retrieval engine in the context of the development of specialized databases holds great promise; however, it is not a trivial task. It requires technical expertise in cloud computing, as well as the development of a unified data-model to which different models can be translated. For example, Pareja-Tobes and colleagues developed the Bio4j framework in which heterogeneous proteomic data is modelled with graphs, stored in a cloud and retrieved using domain specific language implemented in Java [17]. An interesting contribution to cloud-based, federated database development, is the development of BioCloud Search EnGene (BSE), by Dessi and colleagues [18]. BSE is a gene-centric distributed search engine, built upon Google App Engine (GAE). GAE provides a distributed data storage service, which performs distribution, replication and load balancing automatically and supports operations to access objects (i.e. create, read, update, delete) by means of an SQL-like language called GQL.

Here we propose a cloud-based framework of a distributed search engine for biological data. Our framework distributes a query (written in a “google-like” fashion) among several strategic web-based biological databases, such as NCBI’s datasets and Malacards, storing the retrieved results over MongoDB cloud service, and annotating them with the query keywords for future retrieval. Our framework provides a Graphical User Interface, with which the user can explore the retrieved data.

## Implementation

### Framework

**Fig. 1:**
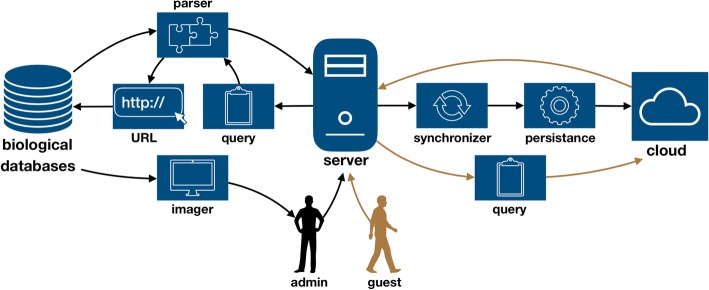
Framework schematic, comprised of server to databases, server to a cloud provider and user to server data streams. System admin can ask the server to execute a distributed search over several databases for a specific entry. The server translates the admin’s request to a query object which is used to define a structured URL. URLs are sent to the appropriate data base, each responds with the requested information, which is parsed to produce a list of corresponding objects. The retrieved objects can be annotated and persisted to a cloud-based specialized database

Our proposed framework is comprised of three main data streams: (I) from a server to web-based databases; (II) from a server to a cloud provider; and (iii) from the user to the server. The construction of a specialized database is administered by an admin user. The admin can ask the server to execute a distributed search over several databases for a specific entry. The server translates the admin’s request to a query object. The query is then translated to a series of structured URLs. Databases often use a fixed URL syntax, which is comprised of a standard set of parameters and retrieval information. The Entrez Programming Utilities, for example, provide a structured URL interface for most NCBI’s databases [19]. Each URL is used to retrieve the query response from a specific source. URLs are sent to the appropriate data base, each responds with the requested information. Since different databases have different data representation, the retrieved information is introduced to a parser – specifically designed for each database - producing a list of objects. The retrieved objects from each database are presented to the admin. If the admin chooses to save the entries to his specialized databases, the server annotates the data with the searched keywords and sends it to a cloud service via a synchronizer, which checks for existing entries, and a persistency agent, which is responsible for data conversion. A ‘guest’ user can search the administered cloud-based specialized database, directly interfacing with the data and exploring results. Schematic of the framework is illustrated in Fig. 1.

### Software description

**Fig. 2:**
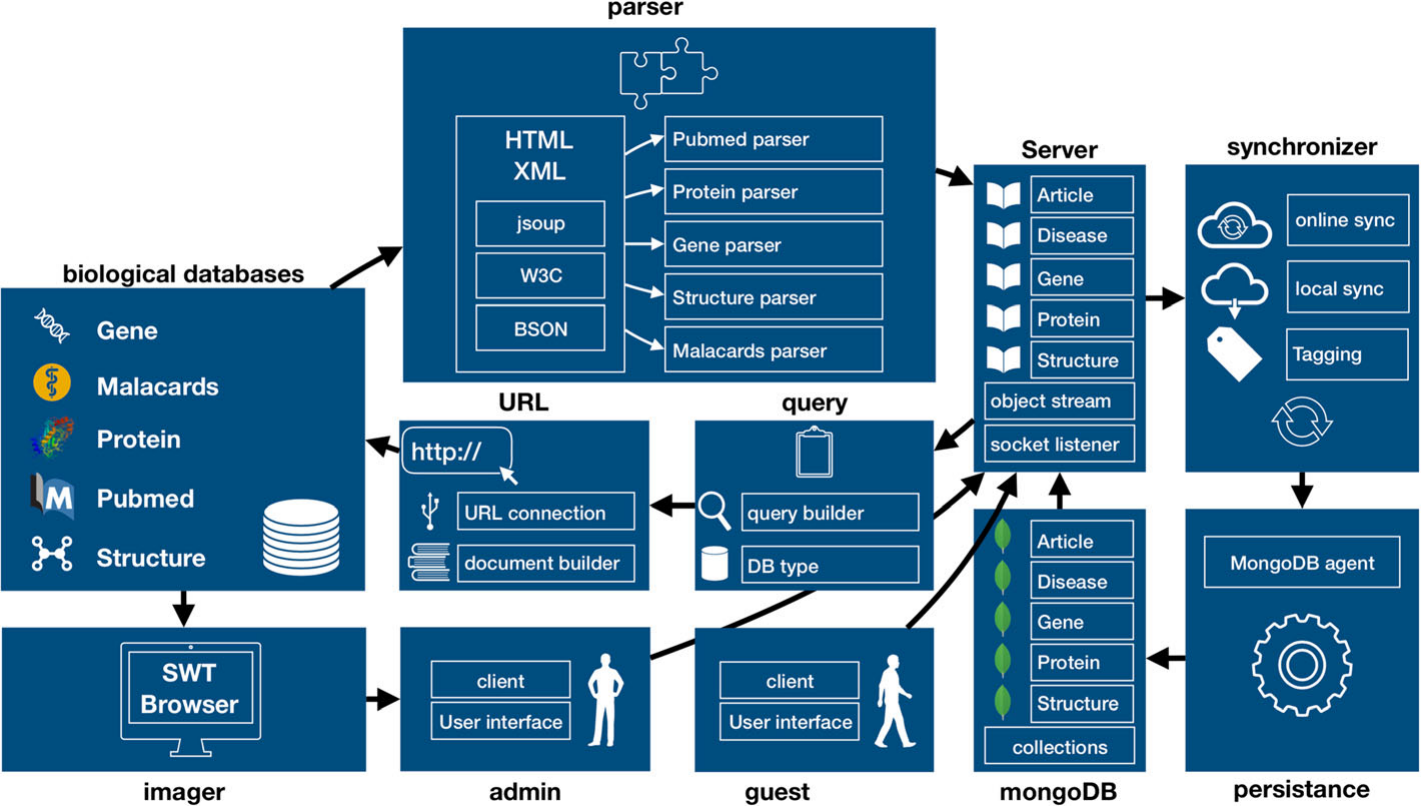
Implementation schematic, comprised of interfaces to online datasets such as Malacards and NCBI’s Gene, Protein, Pubmed and Structure databases, and various processing tools for data parsing

Our proposed framework can be implemented with several different technologies. Here we chose various open-source and free resources for implementation. Java was chosen as the development environment, and MongoDB as the cloud provider. Java was used to create interfaces to online databases such as Malacards and NCBI’s Gene, Protein, Pubmed and Structure databases. Various processing tools were utilized for data parsing. The w4c.dom was used for XML processing, the BSOB library for reading and writing JSON files, Apache Commons’ libraries for CSV parsing, and the jsoup library for HTML processing. The retrieved data is visualized with JAVA’s SWT imaging capabilities. User-server communication was implemented using TCP/IP sockets. A schematic of the implementation is presented in Fig. 2.

The system is comprised of 8 main packages. API packages include the admin and guest packages, each comprised of the appropriate setting parameters, GUI implementation and a communication module. The admin and guest packages make use of specific capabilities, implemented within the ‘hidden’ Persistence, Database and model packages. The server package is also comprised of a GUI and communication capabilities and makes use of all ‘hidden’ packages, including the Parsers and URL packages. UML schematic is shown in Additional file 1: Figure S1.

The admin can initiate a query, defined with a retrieved type, and comprised of an instance of a Database class and a list of Field instances. The database class includes a reference to a specific type of database (which includes all supported databases) and the Field class includes a reference to search fields (e.g. journal, or publication date when the database is Pubmed)**.** UML schematic is shown in Additional file 1: Figure S2. Once a query is defined it is sent to a querybuilder function, where it is translated to the appropriate database-specific structured URL. Following query execution, the retrieved data is analyzed by the appropriate parsers. UML schematic is shown in Additional file 1: Figure S3**.** Each parser creates an object for each instance of the results. For example, the Pubmed parser will create a list of Article objects, the Malacard parser will create a list of disease objects, etc. All created objects implement both Persistable and Serializable interfaces. The persistable interface provides an encapsulation layer, which unifies all objects that are to be persisted in a cloud. The serializable interface allows easy streaming of objects over the communication ports. UML schematic is shown in Additional file 1: Figure S4. Notably, we built a smart data exploration engine that allows the user to interact with the different visualization tools available in our framework. For example, Structure-derived instances can be visually explored via a direct web-based interface to NCBI’s structure viewer, and Gene-derived instances can be similarly explored via NCBI’s sequence explorer.

Persistable objects can be saved to a MongoDB database following tagging and sync, using our MongoDB persistency object. In order to save data over MongoDB, the user has to create a MongoDB account, establishing a link to a specialized database. Account settings can be configured in our framework using a GUI. This account will be used to save as well as to retrieve data from the database. Communication between our framework and the cloud-based database is managed using the persistence package. This package is comprised of a PersistencySetting class, which manages the cloud configuration settings, and a PersistencyAgent. The PersistencyAgent provides a full API to MongoDB allowing for object storing and retrieval. UML schematic is shown in Additional file 1: Figure S5. Data is stored with JSON files. Once data is saved over the cloud it is available for ‘local’ search by guest users. The MongoDB cloud allows for a non-relation-based data storage, which, as previously mentioned, is more appropriate for biological data. Inside the MongoDB, data is organized in collections. Here we initiated 5 collections – each for every type of retrieved data.

For efficient data retrieval from the cloud, data is retrieved in two phases. First, only partial information is displayed (e.g. name and keywords). Following user request the entire instance is retrieved for exploration. This two phases system dramatically reduces the server workload. Moreover, to make searching more informative, a smart tagging mechanism was defined, with which instances of data are tagged with the keywords that were used to retrieve them. When data is displayed for user investigation, it is displayed with these keywords.

### Running example

**Fig. 3:**
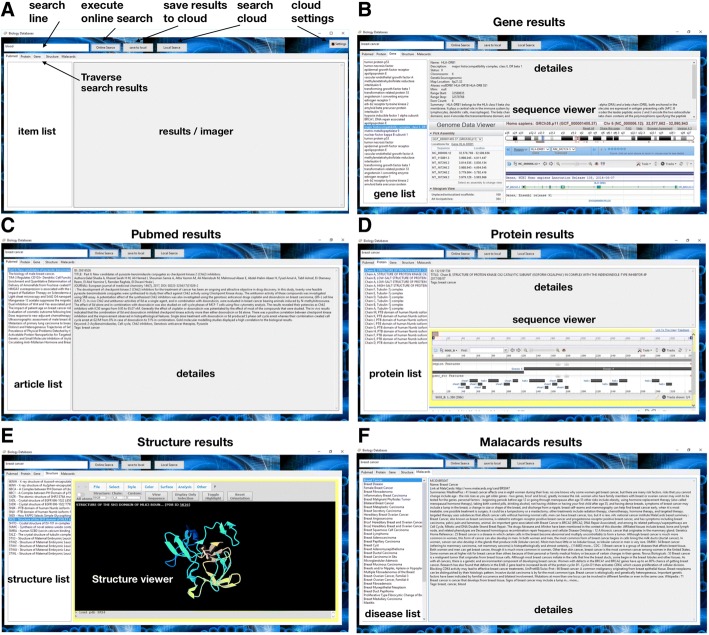
User interface. **a** Graphical user interface. **b**-**f** A running example of a search for ‘breast cancer’

First, the server has to be initiated. Afterwards, either the admin or a guest application may be launched. Since the guest permitted functionalities are a subset of the admin, we will concentrate here on the admin interface. The GUI is shown in Fig. 3a. In the settings menu, the user has to specify the server IP and the URL connection link to a MongoDB account, where the specialized database is to be hosted. The admin application offers an online search, where the user can specify his search openly in a ‘google’ like fashion. At the tab bar, there are several tabs – each for every supported database. Once data is retrieved, results from each database can be explored in the appropriate tab. Let us search for example, the term ‘blood cancer’. Under the Gene tab, a gene list appears. Each gene represents a data instance which was retrieved from NCBI’s Gene database in the first phase of the search. We would choose the ‘*major histocompatibility complex’* and the second phase is initiated, in which all information regarding this data instance is retrieved for exploration. Also, a NCBI’s genome data viewer is initiated and can be used to interactively explore the retrieved sequence (Fig. 3b). Under the Pubmed tab a list of retrieved articles is shown. Choosing an article will enable seeing his title, authors, abstract and other publication details in the details window (Fig. 3c). Under the Protein tab, a list of retrieved proteins is shown. Choosing one of them, such as the ‘Kinase CK II’, will open NCBI’s protein sequence viewer, in which the protein sequence can be interactively explored (Fig. 3d). Similarly, 3-dimensional visualization of protein’s structure can be explored under the Structure tabs with the viewer tool (Fig. 3e), and related diseases can be explored under the Malacard tab (Fig. 3f).

Once the user chooses the ‘save to local’ option, all data is persisted to the MongoDB cloud account and can be monitored via their web interface. For example, the user can monitor the average rate of commands, queries, updated, deletes and inserts. Monitoring example is given in Additional file 1: Figure S6. Moreover, the logical size of the database can be traced in real time (Additional file 1: Figure S7), as well as the number of established connections (Additional file 1: Figure S8), and data-streams (Additional file 1: Figure S9).

The user can explore the stored data in the MongoDB web interface as well. However, data exploration is possible in the free tier subscription only via Mongo Shell (see installation manual for installation and connection details). For example, after connecting to your account you can explore your database with the ‘show dbs’ command:

Select your database using the ‘use’ command and explore the created collections using the ‘show collections’ command:

For example, you can explore the Article collections using the ‘find’ function:

Entries can also be found with JSON-based queries. For example, a specific article can be retrieved using its id:

As was described before, our framework uses smart tagging of data entries, allowing to retrieve data according to the search query which was used to retrieve it. For example:

Detailed description of the MongoDB shell command is given in: https://docs.mongodb.com/v3.4/reference/mongo-shell/. To conclude, we have an integrated environment in which data can be retrieved from multiple databases using our distributed search engine and persisted on a cloud, for future exploration and analysis.

### Software extensions

The above illustrates the architecture used to derive data from online databases and store the retrieved data in a cloud-based specialized data-set. And therefore, being a powerful, easy to use, federated data retrieval engine. However, it is tailored to a specific set of datasets (Malacards, Gene, Protein, Structure and Pubmed), which holds together a tremendous amount of data, but can be extended as needed. More importantly, most specialized databases, being curated by consortiums and labs, must incorporate data which originated from their own work, and not from web-based resources. This framework can therefore be extended in two dimensions: (I) supporting a larger set of databases, and (II) incorporating data from lab work into the larger database. Extending supported databases can be implemented in our proposed framework by creating a parser, a query builder and a data object for this purpose. Our design consists of general support for a data base and query building, encapsulating them with the DataBase and Query classes, which are easily extendable (Additional file 1: Figure S2). As our framework is comprised of various parsing libraries for XML, JSON and HTML documents, they can be easily utilized for the creation of a specific database, as demonstrated for all the databases listed above. Furthermore, as long as the data object implements the Persistable and Serializable interfaces, it can be persisted to the cloud-based database, as illustrated before. The Incorporation of lab-originated data instances in the database is as easy as encapsulating it with an appropriate data class, as mentioned above, and persisting it to memory. Since the proposed framework is freely distributed to the community via Github, we anticipate that the supported list for databases will grow.

## Conclusions

The ever-growing production and coverage of biomedical data introduces challenges in three dimensions (I) the volume (amount of data), variety (types of data), and velocity (speed required for data processing). These 3 dimensions were popularized as the 3Vs of big data. Accordingly, integrating cloud resources and federated data retrieval engine in the context of the development of specialized databases has the potential to enhance the constant development in databases in the biomedical field. Here we propose an extendable, freely distributed framework, allowing for a distributed search among several strategic web-based biological databases, as well as lab-originated data instances over a cloud-based data center. This framework also provides a graphical user interface, with which the user can explore the retrieved data from both online and cloud-based repositories.

While similar frameworks provide integration of some aspects of cloud resources with distributed search (such as the BioCloud Search EnGene), they are primarily focusing on one specific arena (EnGene for example, is focusing on genomic data). Our framework is (1) open source - it can be easily extended to support different niches as well as provide general framework for biodata retrieval and storage and (2) providing a bridge to a cloud database provider. It is unique in the sense that it is based on free community-supported tools, and that it can be extended further if required.

Our framework is distributed under the creative common agreement. To ensure public access to the framework, the source code was uploaded to GitHub at: https://github.com/NBEL-lab/DistCloudBiodata, and it is also accessible via NBEL-lab.com (software). As described above, the framework uses a series of dependable modules, which are freely accessible.

## Additional files

Additional file 1: **Figure S1**. UML schematic of the framework. **Figure S2**. UML schematic of the Query and DataBase classes. **Figure S3**. UML schematic of the parsing classes. **Figure S4**. UML schematic of the data classes. **Figure S5**. UML schematic of the persistency classes. **Figure S6**. MongoDB cloud commands monitor. **Figure S7**. MongoDB cloud storage monitor. **Figure S8**. MongoDB cloud connection monitor. **Figure S9**. MongoDB cloud data-streams monitor. (PDF 506 kb)
