# Supplementary material for: Distributed retrieval engine for the development of cloud-deployed biological databases
Source: BioData Min. 2018 Nov 12;11:26. doi: 10.1186/s13040-018-0185-5 (PMC6233384; doi:10.1186/s13040-018-0185-5)
Supplement: Supplementary file 1 — Figure S1. UML schematic of the framework. Figure S2. UML schematic of the Query and DataBase classes. Figure S3. UML schematic of the parsing classes. Figure S4. UML schematic of the data classes. Figure S5. UML schematic of the persistency classes. Figure S6. MongoDB cloud commands monitor. Figure S7. MongoDB cloud storage monitor. Figure S8. MongoDB cloud connection monitor. Figure S9. MongoDB cloud data-streams monitor. (PDF 506 kb) [file 13040_2018_185_MOESM1_ESM.pdf]

Distributed retrieval engine for the development of cloud-deployed biological databases

David Buzaglo[1], Israel Chasida[1], Elishai Ezra Tsur[1*]

[1] Neuro-biomorphic Engineering Lab, Jerusalem College of Technology, Israel

[*] Correspondence should be addressed to: elishai@nbel-lab.com

Supplementary Information

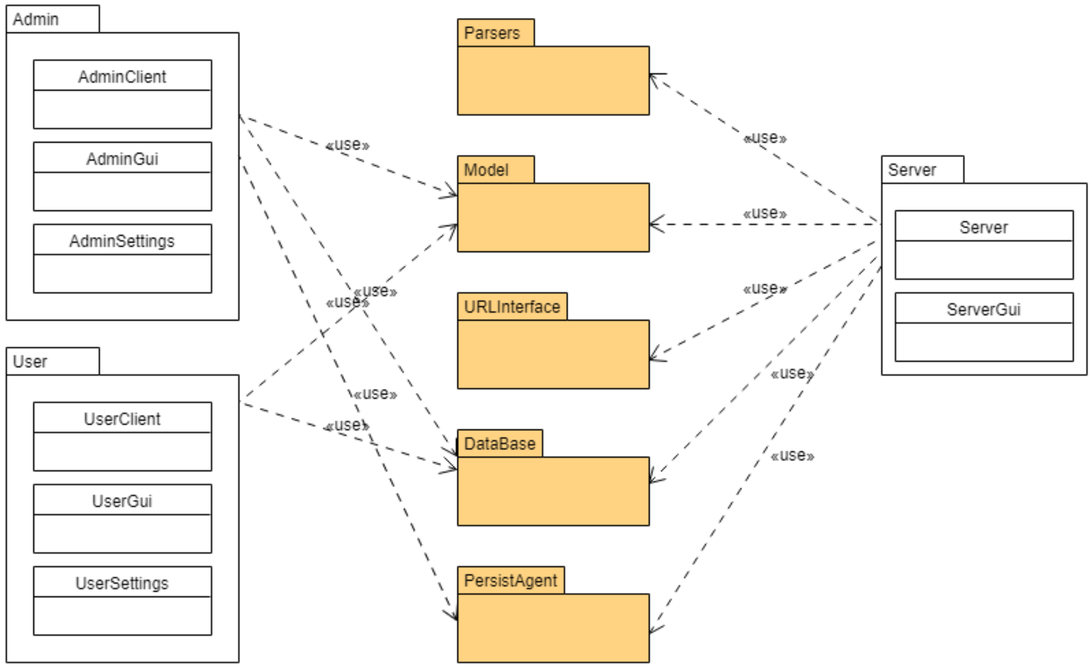

Figure S1. UML schematic of the framework

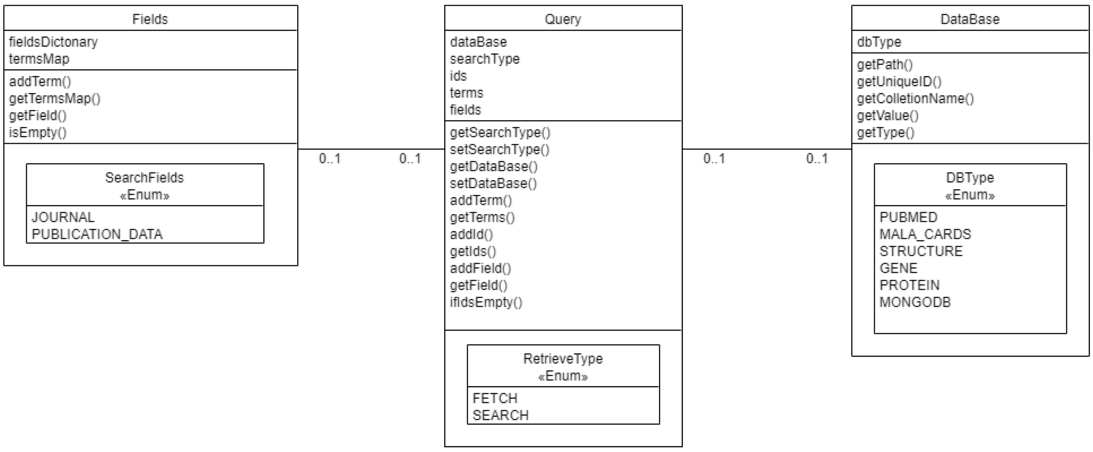

Figure S2. UML schematic of the Query and DataBase classes

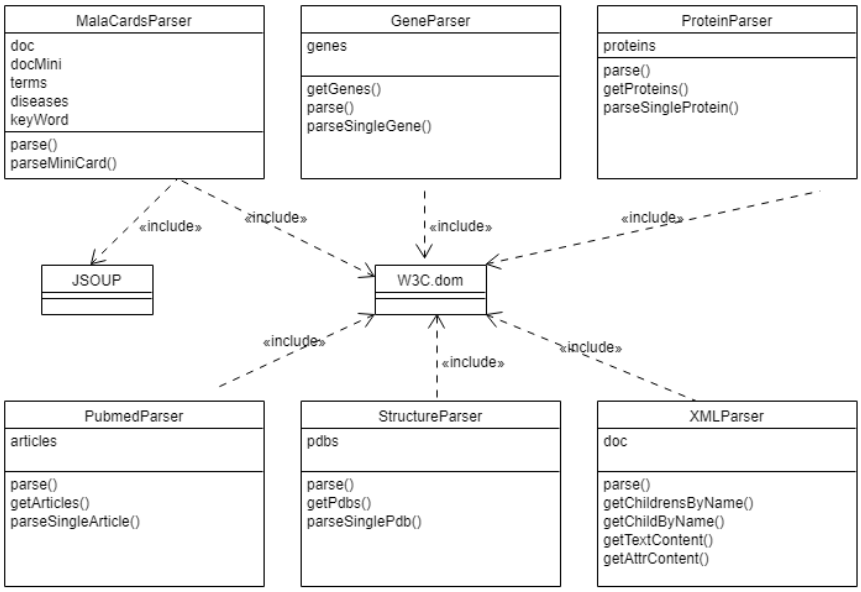

Figure S3. UML schematic of the parsing classes

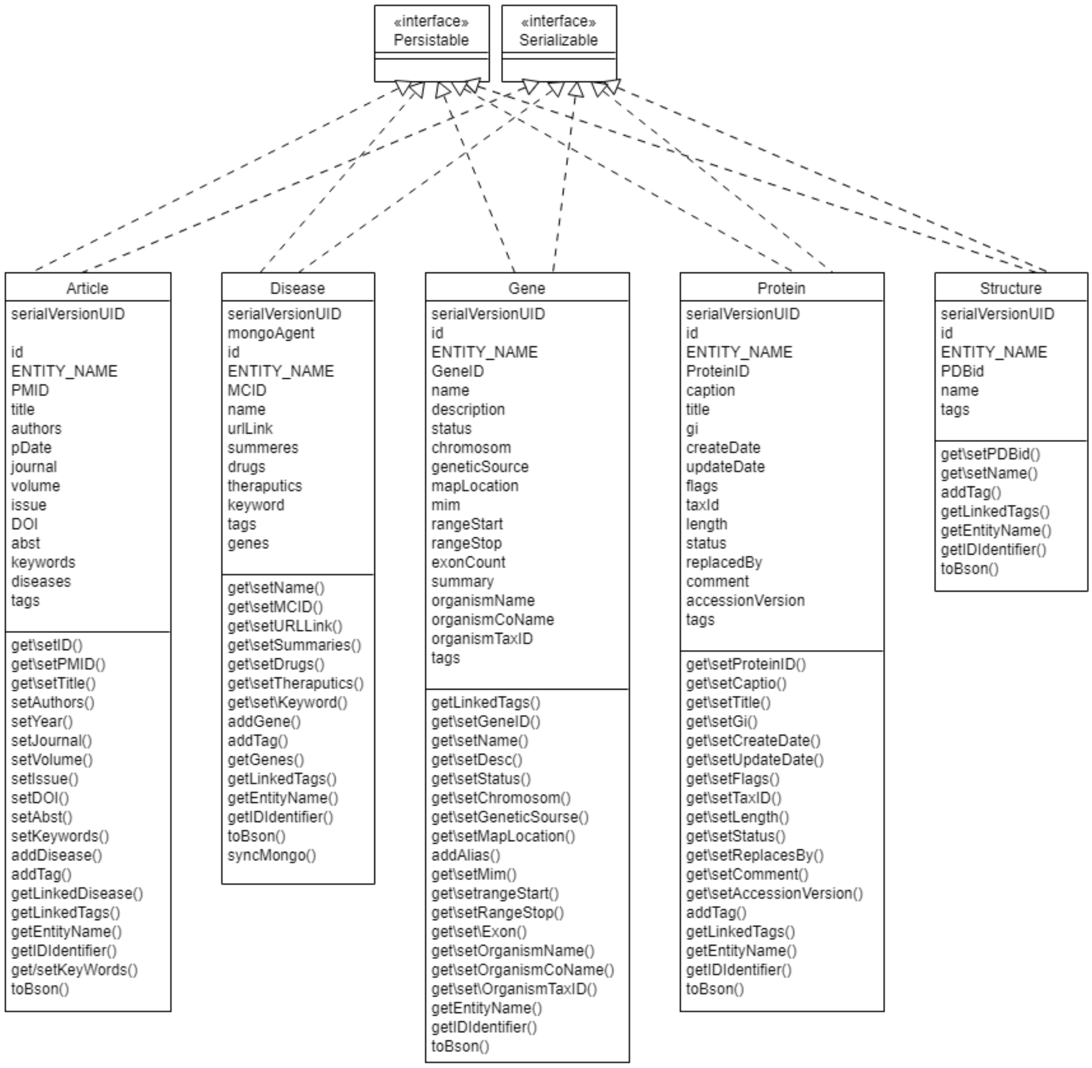

Figure S4. UML schematic of the data classes

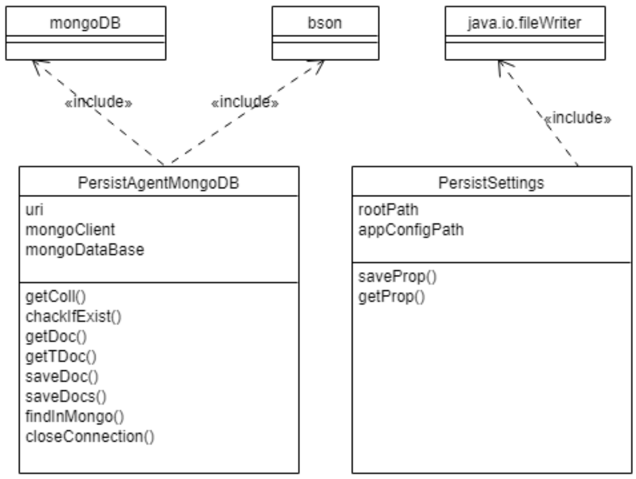

Figure S5. UML schematic of the persistency classes

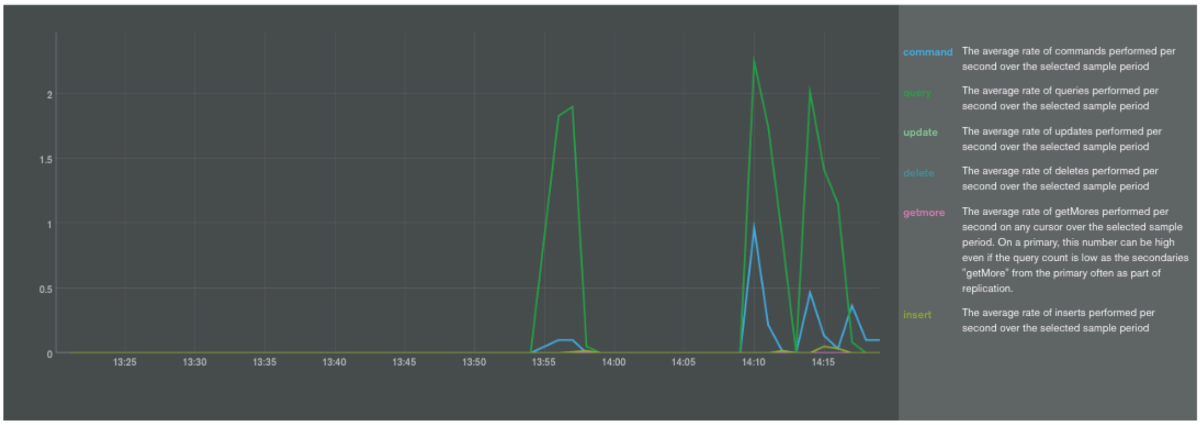

Figure S6. MongoDB cloud commands monitor.

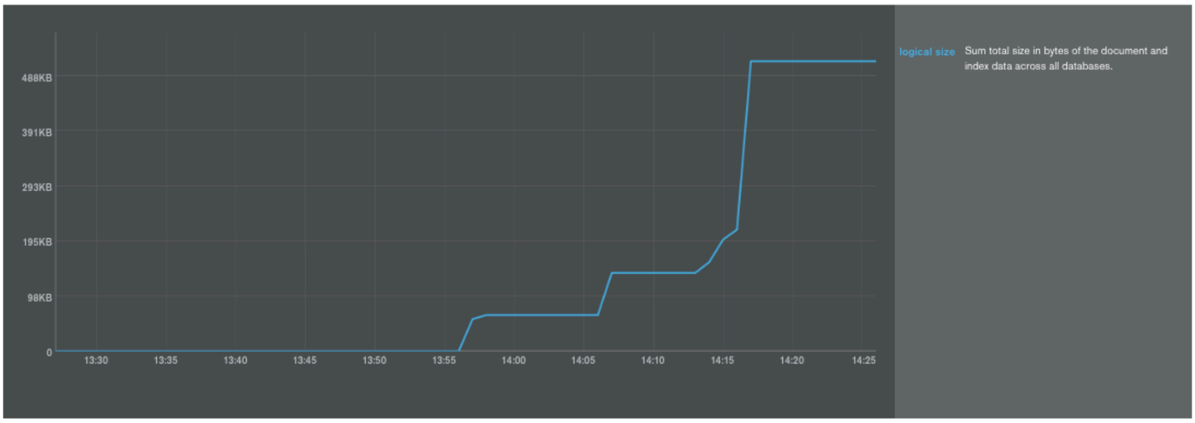

Figure S7. MongoDB cloud storage monitor.

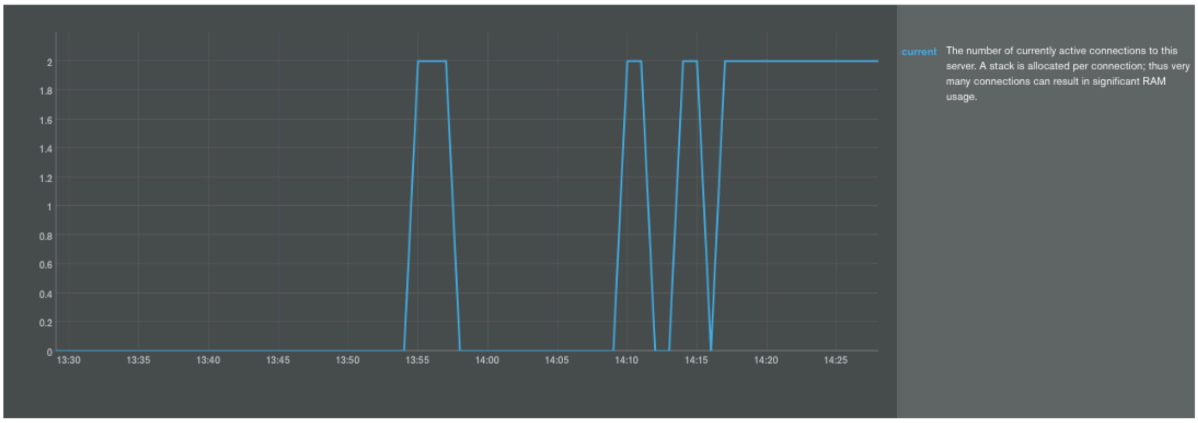

Figure S8. MongoDB cloud connection monitor.

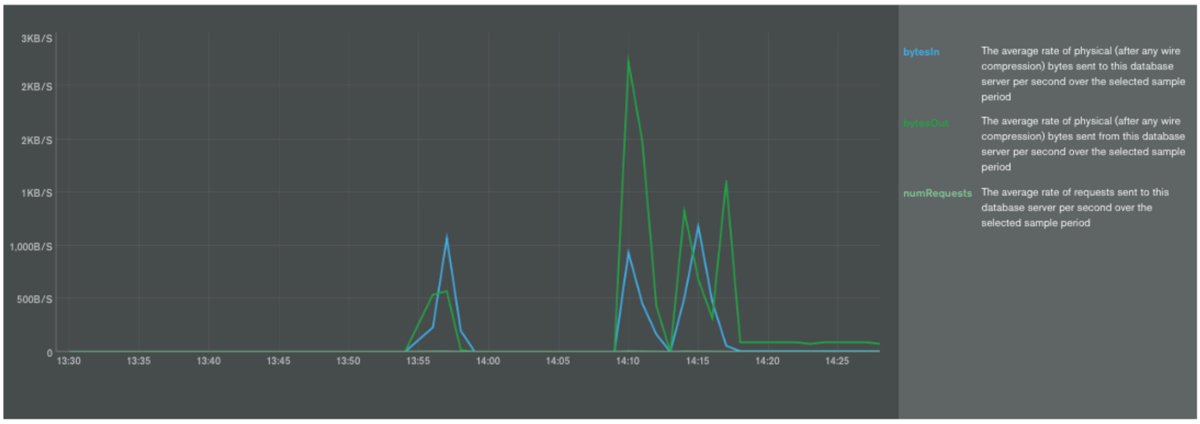

Figure S9. MongoDB cloud data-streams monitor.
